# Resignation of Prof. Warren

**Published:** 1847-04

**Authors:** 


ARTICLE VIII.

### RESIGNATION OF PROF. WARREN.

Prof. Warren who has, with great distinction to himself and the school, occupied the chair of anatomy and physiology in the medical department of Harvard University, at Boston, for forty years, has tendered his resignation and retired from its arduous duties.

On taking leave of the class he delivered an address said to abound in good feeling and sound advice. He has been elected Emeritus Professor of Anatomy and Surgery. And Oliver W. Holmes, M. D., is appointed his successor.

